# Bromocriptine use for sudden peripartum cardiomyopathy in a patient with preeclampsia: a case report

**DOI:** 10.1186/s40981-019-0256-8

**Published:** 2019-06-07

**Authors:** Saya Hakata, Takeshi Umegaki, Takehiro Soeda, Kota Nishimoto, Akiko Ando, Natsuki Anada, Takeo Uba, Chisato Sumi, Takahiko Kamibayashi

**Affiliations:** Department of Anesthesiology, Kansai Medical University Hospital, 2-3-1 Shin-machi, Hirakata, Osaka 573-1191 Japan

**Keywords:** Peripartum cardiomyopathy, Preeclampsia, Bromocriptine

## Abstract

**Background:**

Peripartum cardiomyopathy is an uncommon form of heart failure that occurs in otherwise healthy women during pregnancy or until 5 months postpartum. Here, we report a rare case where a female patient underwent cesarean section after the occurrence of preeclampsia and intrauterine fetal death, and developed peripartum cardiomyopathy following postsurgical respiratory distress. The prompt initiation of inotropic drug and bromocriptine therapy quickly restored cardiac function.

**Case presentation:**

The patient was a 36-year-old woman who underwent emergency cesarean section for a previous preeclampsia and an intrauterine fetal death that occurred after 24 weeks of pregnancy. In addition, the patient had an extremely low platelet count of 5000/μL on admission. She had been diagnosed as idiopathic thrombocytopenic purpura at the age of 29 years old and treated with prednisolone at 15 mg/day. Therefore, the cesarean section was performed under general anesthesia. The patient did not exhibit respiratory or hemodynamic dysfunction during surgery. However, she developed respiratory distress with sinus tachycardia after extubation and was transferred to the intensive care unit. A chest radiograph showed butterfly shadows, and transthoracic echocardiogram confirmed the reduction of left ventricle contractility (ejection fraction 20%). She was diagnosed with peripartum cardiomyopathy and treated immediately with intravenous milrinone, oral bromocriptine, and angiotensin-converting enzyme inhibitor. Respiratory and hemodynamic function improved rapidly, and the patient was moved to the general ward 2 days after surgery. Fourteen days after surgery, the patient had an ejection fraction of 57%. The patient recovered without any further complications and was discharged 24 days after surgery.

**Conclusion:**

A sudden case of peripartum cardiomyopathy was successfully managed by a prompt diagnosis and treatment with inotropic agents and bromocriptine.

## Background

Peripartum cardiomyopathy (PPCM) refers to an uncommon form of idiopathic heart failure that occurs during pregnancy or until 5 months postpartum [1, 2]. While some patients experience favorable clinical outcomes, serious cases require assisted circulation and cardiac function can deteriorate to the point that a heart transplant is necessary. Here, we report a rare case where a female patient underwent cesarean section after the occurrence of intrauterine fetal death, and suddenly developed postsurgical respiratory distress and acute heart failure. She was diagnosed with PPCM, and the administration of bromocriptine therapy quickly and successfully restored cardiac function.

## Case presentation

**Fig. 1:**
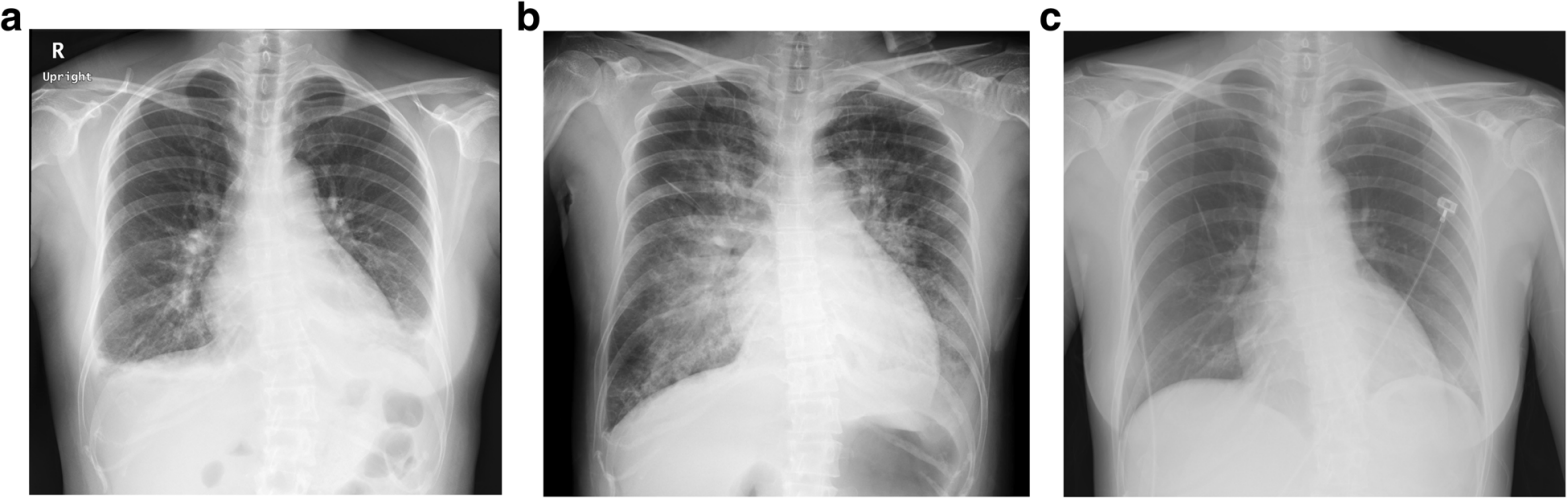
Chest radiograph shows bilateral pleural effusion without pulmonary edema before surgery (**a**), butterfly shadows on the day of surgery (**b**), and the disappearance of the butterfly shadows on postoperative day 4 (**c**)

**Fig. 2:**
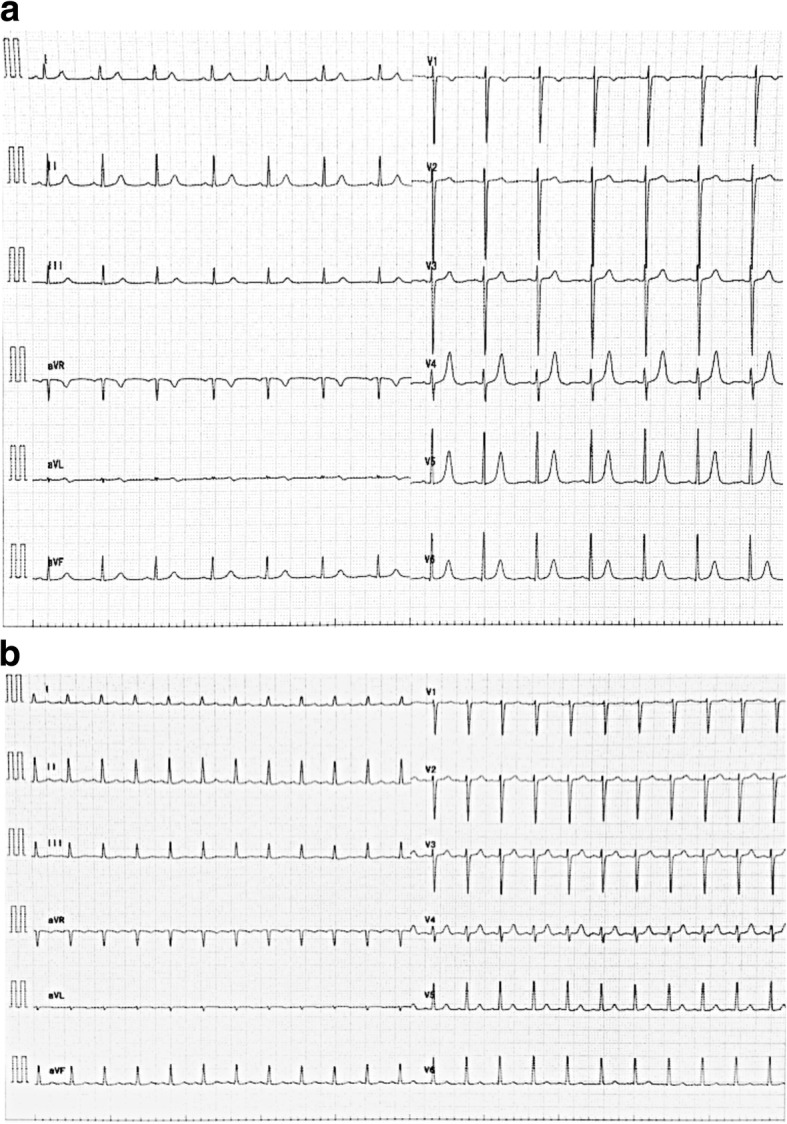
The patient’s 12-lead electrocardiograms showing sinus rhythm and no sign of ST-T change before surgical operation (**a**) and immediately after admission to a coronary care unit (**b**)

A 36-year-old woman, who had had a previous cesarean section, became pregnant by in vitro fertilization. The patient had been diagnosed as idiopathic thrombocytopenic purpura at the age of 29 years old that was being treated with oral prednisolone (15 mg/day). But she had no past history of hypertension and diabetes mellitus. Her blood pressure before 24 weeks of gestation was less than 120 mmHg for systolic pressure and a diastolic level of less than 80 mmHg. Also, proteinuria was not present. Intrauterine fetal death without the development of fetal hydrops occurred after 24 weeks of pregnancy. At the same time, she was diagnosed as preeclampsia with a blood pressure of 160/123 mmHg, albuminuria (> 1000 mg/dL), and generalized edema on admission. Therefore, she was immediately scheduled for emergency cesarean section. A chest radiograph (Fig. 1a) revealed blunting of the lateral costophrenic angle, which indicates that there was small pleural effusion of soft tissue, and the cardiothoracic ratio (CTR) was 49.4%. Pulmonary edema was not evident in the chest radiograph (Fig. 1a). The electrocardiogram showed sinus rhythm without ST-T changes (Fig. 2a). As her platelet count was extremely low at 5000/μL, she received 20 units of platelets in Japan before the cesarean section was performed under general anesthesia. The C-reactive protein (0.833 mg/dL) and creatine kinase levels (63 IU/L) were within the normal range. She took medication of 20 mg of oral prednisolone before the surgical operation.

Upon entry to the operating room, the patient showed blood pressure of 109/59 mmHg, a pulse of 121 bpm, and a peripheral oxygen saturation (SpO_2_) of 97% under 2.0 L/min supplemental oxygen via nasal cannula. General anesthesia was induced using propofol (120 mg), rocuronium (50 mg), and fentanyl (200 μg). During operation, she was managed with her systolic blood pressure from 90 to 120 mmHg, heart rate from 100/min to 120/min, and PaO2/FiO2 ratio ≥ 300 under fraction of inspiratory oxygen of 0.40. The duration of surgery and anesthesia was 52 min and 105 min, respectively. Intraoperative blood loss was 1281 mL including amniotic fluid, and 800 mL of crystalloid and 500 mL of colloid were administered. After the completion of the surgery, the trachea of the patient without intraoperative excessive tachycardia, low blood pressure, low oxygenation, and pink frothy sputum was extubated in the operating room. But she gradually developed respiratory distress with sinus tachycardia (120–140/min) and slightly high systolic blood pressure (140–150 mmHg) right after extubation. Her SpO_2_ decreased to 91% despite supplemental oxygen at 6 L/min. As the use of a face mask was found to improve her SpO_2_ levels, the patient was promptly transferred to the general intensive care unit while wearing the mask. Our general intensive care unit is managed as closed-intensive care unit by the anesthesiologists. Therefore, we anesthesiologists started treatment immediately.

The patient was started on noninvasive positive pressure ventilation (NIPPV). Respiratory distress was alleviated, and the fraction of inspired oxygen was reduced. A chest radiograph was obtained after the patient was admitted to the intensive care unit, which showed the presence of butterfly shadows (Fig. 1b). The CTR was 57.1%, indicating cardiomegaly. The electrocardiogram revealed sinus tachycardia without ST-T change (Fig. 2b), and therefore, ischemic heart disease was not suspected. We strongly suspected an acute left ventricular failure with a New York Heart Association class IV. Transthoracic echocardiogram (TTE) confirmed the reduction of left ventricle contractility with an end-diastolic internal diameter of 40 mm, an end-systolic internal diameter of 37 mm, and an ejection fraction (EF) of 20%. The left ventricle exhibited diffuse severe hypokinesis without hypertrophy. Tricuspid regurgitation represented trivial and peak pressure gradient of 16 mmHg. TTE did not show Takotsubo cardiomyopathy-like left ventricular dysfunction. The patient had no previous history of heart disease, and a review of the clinical course led to a diagnosis of PPCM. As a result, the patient was moved to the coronary care unit for further treatment.

Milrinone (0.25 μg/kg/min) was administered through continuous intravenous infusion to restore myocardial contractility. The anti-prolactin bromocriptine (5 mg/day) and angiotensin-converting enzyme inhibitor (enalapril maleate) were administered orally. The patient showed rapid improvement in respiratory and hemodynamic function after beginning therapy. The patient well preserved renal function. Therefore, there was no need for carperitide or other diuretics. On the second postoperative day, the patient was moved to the general ward with a blood pressure of 140/80 mmHg, an SpO_2_ of 97% (supplemental oxygen at 2 L/min), and an EF of 40%. Milrinone was discontinued on the fourth postoperative day. At that time, the patient had a CTR of 51.1%, and a chest radiograph confirmed that the butterfly shadows had disappeared (Fig. 1c). Fourteen days after surgery, TTE showed that the patient had an EF of 57%. Coronary computed tomography did not indicate any stenotic lesions in the coronary arteries.

Table 1 shows the time-dependent changes in prolactin and N-terminal pro-brain natriuretic peptide (normal range 0–125 pg/mL) after the start of bromocriptine administration. The patient was administered oral bromocriptine at a dose of 5 mg/day for 14 days, which resulted in a reduction in prolactin levels from 228 to 3.4 ng/mL. The bromocriptine dose was reduced to 2.5 mg/day for another 2 weeks before therapy was discontinued. The patient recovered without any further complications and was discharged 24 days after surgery.

**Table 1:** Time course of prolactin and NT-proBNP levels after the start of bromocriptine administration

	Day of surgery	Postoperative day 1	Postoperative day 2	Postoperative day 13
PRL (ng/mL)	228	20.6	13.1	3.4
NT-proBNP (pg/mL)	16,769	4911	1978	51

*Abbreviations*: *PRL* prolactin, *NT-proBNP* N-terminal pro-brain natriuretic peptide

## Discussion

Women may develop PPCM during pregnancy or up to 5 months after giving birth despite having no prior history of heart disease. In Japan, PPCM is reported to occur once every 20,000 deliveries [3], which is markedly lower than the mean incidence of patients in Asians in the USA [4]. Although the low incidence in Japan may be due to the provision of excellent perinatal care, it is also possible that there is a generally low awareness toward this condition among physicians, thereby leading to infrequent reports [5]. A recent study estimated that PPCM is associated with a mortality rate of 4% in Japan and that 53% of cases experience improvement of the left ventricular EF to 50% or more [6].

Diagnostic criteria for PPCM have been previously proposed [1, 7]. These criteria include left ventricular EF < 45–55%, fractional shortening < 30%, the lack of any previous history of heart disease, and the lack of other potential causes of heart failure. In this way, PPCM diagnoses are dependent on exclusion criteria that preclude other identifiable causes of heart failure in women without any pre-existing heart disease [7]. The differential diagnosis of potential PPCM cases should include ischemic cardiomyopathy, dilated cardiomyopathy, and myocarditis. Ischemic cardiomyopathy can be excluded by testing for increased cardiac enzymes in blood, detecting characteristic ST-T changes in electrocardiograms, and the use of TTE. However, dilated cardiomyopathy and PPCM are similarly characterized by diffuse systolic dysfunction and ultrasound-detected left ventricular dilatation, which complicates their distinction. The careful examination of family and patient histories is therefore particularly important for PPCM diagnoses. Preeclampsia has been reported to occur in 30% of PPCM cases [8] and should therefore be included as a potential indicator of PPCM as part of the differential diagnostic process. In practice, however, it is difficult to suspect PPCM with myocardial dysfunction based solely on preeclampsia. The patient described in this report had presented with left ventricular enlargement prior to surgery, and the observed generalized edema was attributed to preeclampsia. We did not suspect heart failure until the chest radiography was performed postoperatively.

Bromocriptine is reportedly effective in the treatment of cardiomyopathy [9–12]. It has been hypothesized that the over-secretion of prolactin increases the quantity of a 16-kDa antiangiogenic prolactin fragment, which may be a key pathological mediator of PPCM through the impairment of myocardial microvacularization [9]. The administration of bromocriptine, an anti-prolactin, may therefore reduce the quantity of this prolactin fragment and facilitate the restoration of cardiac function [9].

The patient did not receive nifedipine or magnesium sulfate before cesarean section, because the patient had no sign of eclampsia and her blood pressure was going to fluctuate around 160/110 mmHg. However, a recent study reported that blood pressure requires urgent treatment in a monitored setting when severe (> 160/110 mmHg) [13]. In this case, pulmonary congestion right after cesarean section might be mainly caused by increased vascular permeability and elevated blood pressure. Thus, the patient might show rapid improvement. Therefore, the use of oral or intravenous antihypertensive drugs should be considered. In addition, it might have been better to perform echocardiography on admission to confirm a potential PPCM in the patients with preeclampsia.

## Conclusions

Here, we report the case of a patient who suddenly developed acute PPCM after undergoing a cesarean section, but a prompt diagnosis and treatment with inotropic agents and bromocriptine allowed her to regain cardiac function. Although PPCM remains a relatively rare condition, it should be included in the differential diagnosis of patients who present with preeclampsia.

## Data Availability

Please contact the corresponding author for data requests.

## Abbreviations

CTR: Cardiothoracic ratio
EF: Ejection fraction
NIPPV: Noninvasive positive pressure ventilation
PaO2/FiO2 ratio: The ratio of partial pressure arterial oxygen and fraction of inspired oxygen
PPCM: Peripartum cardiomyopathy
SpO_2_: Peripheral oxygen saturation
TTE: Transthoracic echocardiogram
